# Neuroprotective Effect of a DJ-1 Based Peptide in a Toxin Induced Mouse Model of Multiple System Atrophy

**DOI:** 10.1371/journal.pone.0148170

**Published:** 2016-02-22

**Authors:** Micaela Johanna Glat, Tali Ben-Zur, Yael Barhum, Daniel Offen

**Affiliations:** 1 Sagol School of Neuroscience, Tel-Aviv University, Tel Aviv, Israel; 2 Felsenstein Medical Research Center, Sackler School of Medicine, Tel-Aviv University, Petach Tikva, Israel; National Institutes of Health, UNITED STATES

## Abstract

Multiple System Atrophy (MSA) is a sporadic neurodegenerative disorder characterized by parkinsonism, cerebellar ataxia and dysautonomia, in various combinations. In MSA with parkinsonism (MSA-P), the degeneration is mainly restricted to the substantia nigra pars compacta and putamen. Studies have identified alterations in DJ-1 (PARK7), a key component of the anti-oxidative stress response, in Parkinson’s disease (PD) and MSA patients. Previously we have shown that a short DJ-1-based peptide named ND-13, protected cultured cells against neurotoxic insults and improved behavioral outcome in animal models of Parkinson’s disease (PD). In this study, we used the 3-Nitropropionic acid (3-NP)-induced mouse model of MSA and treated the animals with ND-13 in order to evaluate its therapeutic effects. Our results show that ND-13 protects cultured cells against oxidative stress generated by the mitochondrial inhibitor, 3-NP. Moreover, we show that ND-13 attenuates nigrostriatal degeneration and improves performance in motor-related behavioral tasks in 3-NP-treated mice. Our findings suggest a rationale for using ND-13 as a promising therapeutic approach for treatment of MSA.

## Introduction

Multiple System Atrophy (MSA) is a sporadic neurodegenerative disease affecting 4–5 people per 100,000 individuals. The disease is most often diagnosed after the age of 60, with a mean survival of 6–9 years after the diagnosis [1], [2]. MSA is classified as two subtypes: MSA with Parkinsonism (MSA-P), and MSA with cerebellar ataxia (MSA-C). In MSA-P the degeneration is observed mainly in the striatum and the substantia nigra, with the principal symptoms being bradykinesia and tremors. In MSA-C the cerebellum is the main locus of neurodegeneration, and the principal symptom is poor coordination [3]. These two MSA subtypes share the same autonomic dysfunctions that correlate with neurodegeneration in the autonomic brain stem centers, intermediolateral cell columns, and Onuf’s nucleus in the spinal cord [4], [5].

Mitochondrial dysfunction and oxidative stress have been implicated in the onset of several neurodegenerative diseases [6], [7]. Epidemiological studies of MSA have suggested that exposure to pesticides, insecticides, or chemicals alternating the mitochondrial function may be associated with increased risk of MSA pathology [8], [9]. Furthermore, several studies have directly linked oxidative stress to MSA: In transgenic mouse models of MSA oxidative stress induced or augmented neurodegeneration, motor deficits and enhanced oxidative modifications of alpha-synuclein [10], [11]. In addition, an increase in oxidative stress markers was observed in the CSF of MSA patients [12].

DJ-1 is a ubiquitous redox-responsive cytoprotective protein that protects against oxidative and neurotoxic insults [13–18]. DJ-1 has numerous functions: preserving mitochondrial function, regulating redox signaling kinase pathways and acting as a transcriptional regulator thereby affecting anti-oxidant genes and dopamine homeostasis [19–22]. DJ-1 mutations are known to cause early onset autosomal recessive PD [23], and it is abundantly expressed in reactive astrocytes of both PD and MSA patients [24]. In addition, loss of DJ-1 stability and cytoprotecitive function cause microtubule retraction in a cell culture model of MSA [25]. We have previously shown that a DJ-1 based peptide, ND-13, protects against glutamate and SIN-1 induced toxicity in a mouse model of ALS [26]. Additionally, we demonstrated that ND-13 attenuated dopaminergic system dysfunction and improved the behavioral outcome observed in the 6-hydroxydopamine and in the 1-methyl-4-phenyl-1,2,3,6-tetrahydropyridine mouse models of PD [27].

In the present study we evaluate the effect of ND-13 in toxin- *in-vitro* and *in-vivo* models of MSA-P. We show that ND-13 protected cultured cells against 3NP-induced oxidative stress and improved their survival rates. Moreover, 3NP-exposed mice treated with ND-13 exhibited decreased inflammation, improved motor function and reduced neuronal loss in the striatum in comparison to the control groups. These finding suggest a possible therapeutic effect of ND-13 on patients afflicted with MSA.

## Materials and Methods

### DJ-1 Based Peptide: ND-13

ND-13 is a 20 amino acids peptide, composed of 13 amino acids based on the DJ-1 protein attached to a TAT-derived 7 amino acids peptide (CPP) designed to improve cell penetration and facilitate the delivery into the central nervous system [28], [29]. The complete sequence of ND-13 with the CPP is: YGRKKRRKGAEEMETVIPVD [27]. As a negative control, we used a scrambled peptide, composed of the same 13 amino acids but in a reversed order, attached to the same 7 amino acids CPP moiety.

### Exposure of Cells to 3-NP and Cell Viability Measurements

Rat adrenal medullary cell-line, PC-12, were obtained from ATCC (Rockville, USA). Cells were plated in 96 well plates at 20,000 cells per well in 100 μl DMEM supplemented with 100 mg/ml streptomycin, 100 U/ml penicillin, 12.5 units/ml nystatin, 2 mM L- glutamine, and 10% fetal calf serum (Biological Industries). After 24 hours, cells were treated with PBS, ND-13 (3 μM, 6 μM, 12.5 μM, 25 μM and 50 μM) or with the scrambled peptide (12.5μM and 50μM). Following 1 hour of incubation, cells were exposed to 3-NP (80 mM) for 24 h, at the end of which, Alamar blue 10% (AbD Serotec) was added to the cells for an additional 3 hours. Cell viability was assessed by measuring fluorescence intensity at an excitation wavelength at 560 nm and an emission wavelength at 590 nm using a fluostar device (Synergy HT, BioTeck Instruments, Inc.).

### Measurements of Mitochondrial Membrane Potentials (ΔΨm)

Mitochondrial transmembrane potential was determined by using the fluorescent probe tetramethylrhodamine ethyl ester (TMRE, Abcam). PC12 cells were plated in 6 well plates at 100,000 cells per well. Cells were treated with ND-13 at a concentration of 10μM and after 1 hour of incubation, cells were exposed to 120mM of 3-NP and incubated with the toxin for an additional 5 hours. In order to assess the mitochondrial transmembrane potential, TMRE (100 nM) was added to the cells for 20 minutes following which, fluorescence levels were measured using fluorescence-activated cell sorter (FACS, Beckman Coulter). The data was analyzed with Kaluza software.

### Treatments in the Toxin Induced Mouse Model of MSA

Eight-week-old male C57bl mice (Harlan, Jerusalem) were placed under 12-hour-light/12-hour-dark conditions and housed in individually ventilated cages (IVC) with free access to food and water. All experimental procedures were approved by the Tel Aviv University Committee of Animal Use for Research and Education. Every effort was made to reduce the number of mice used and to minimize their suffering. Twenty seven animals were randomly assigned to one of two groups: ND-13 (n = 14) and saline (n = 13). They were then positioned in a stereotaxic frame and anesthetized with ketamine and xylazine (100 mg/kg and 8 mg/kg, respectively). Striatonigral denervation was induced by stereotactic injections of 0.5 μl of 3-NP (at a concentration of 70 mg/ml in normal saline) into the right striatum (anterior +0.5 mm; lateral −2 mm; ventral −2.7 mm, as determined from the bregma and the skull surface).

ND-13 (3 mg/kg) or saline were administered systemically by subcutaneous injections, three hours before, and again one hour after 3-NP injection, and then twice a day over four consecutive days.

### Behavioral Test and Analysis

1. Amphetamine and apomorphine induced asymmetrical rotation: Mice were tested for apomorphine and amphetamine-induced turning behavior 14 and 18 days after the 3-NP injection. Motor behavior was recorded by video tracking systems, EthoVision XT (Noldus), for 60 minutes after subcutaneous injection of apomorphine (10 mg/kg) or amphetamine (5 mg/kg). The net rotation asymmetry score for each test was calculated by subtracting ipsilateral turns from the turns contralateral to the lesion.
2. Cylinder test: Motor asymmetry was measured by the cylinder test one week after 3-NP injection. The number of wall contacts with each forelimb when rearing was computed. Animals that did not meet the criterion of 15 rearing cycles were excluded from this assay. The cylinder test score was determined by calculating the use of the affected contralateral forepaw–intact ipsilateral forepaw /total (contralateral + ipsilateral + both).
3. Balance beam: The test assesses motor coordination and balance. The balance beam was constructed as previously described [4]. Mice were trained over three consecutive days. On the first day animals were habituated to the goal box for 5 min. On the second and third day mice were trained to enter the goal box initially from a position directly adjacent to the box. On the fourth day mice were placed on the extreme end of the beam in the start area and facing away from the beam. Each test consisted of five consecutive measurements of the time it takes each animal to cross the beam and to get into the goal box.
4. Pole test: To assess the mouse locomotor activity the mice were placed head-upward on the top of a rough-surfaced vertical pole, 1 cm wide and 50 cm high [9]. The time until it descends to the floor was recorded in 4 trials.

### RNA Extraction and Real Time PCR

RNA extraction, cDNA synthesis and real time PCR were done as previously reported [20]. The primers used are Interferon Gamma (INF-gamma) forward: 5’ -TGA ACG CTA CAC ACT GCA TCT TGG-3’ and reverse: 5’ -CGA CTC CTT TTC CGC TTC CTG AG-3’.

### Protein Extraction and Western Blotting

Whole cell protein extraction and Western blotting were performed as previously reported [20]. Membranes were probed with anti DARPP-32 (1:1000, Abcam), TH (1:2000, Sigma), GFAP (1:1000, Invitrogen) and anti beta-actin (1:2000, Millipore). Visualization and analysis of band intensities were performed using the Odyssey system (LICOR, Lincoln, NE, USA).

### Tissue Processing and Histology

Mice were anesthetized and perfused with PFA 4%. Brains were dissected, embedded in optimal cutting temperature (OCT, Tissue-Tek) and sectioned (10 μm) using a cryostat. For immunohistochemistry, slides underwent citrate buffer antigen retrieval and were then incubated with blocking solution (5% goat serum, 1% BSA, 0.05% Triton-X in PBS) for 1 hr. Then, slides were incubated overnight at 4°C with the following primary antibodies: rabbit anti -GFAP (1:200, Invitrogen), rabbit anti-DARPP-32 (1:50, Abcam), and mouse anti-TH (1:200, Sigma). Then, sections were incubated with the secondary antibody Alexa Fluor 488 and 568 (1:500; Invitrogen) for 1 hr. The nuclei were counterstained with 4, 6-diamidino-2-phenylindole (DAPI; 1:1000; Sigma-Aldrich).

### Statistical Analysis

Statistical analysis of data sets was carried out with the aid of GraphPad Prism for Windows (Graphpad Software, CA, USA). The results are expressed as means ± standard error (SE). Comparisons of two groups were conducted using a 2- tailed Student’s t test. Statistical analyses for three or more groups were performed using one or two-way-ANOVA followed by post hoc comparison. A significance level of p≤0.05 was set for all statistical tests.

## Results

### ND-13 Protects PC12 Cells Against 3-NP

**Fig 1:**
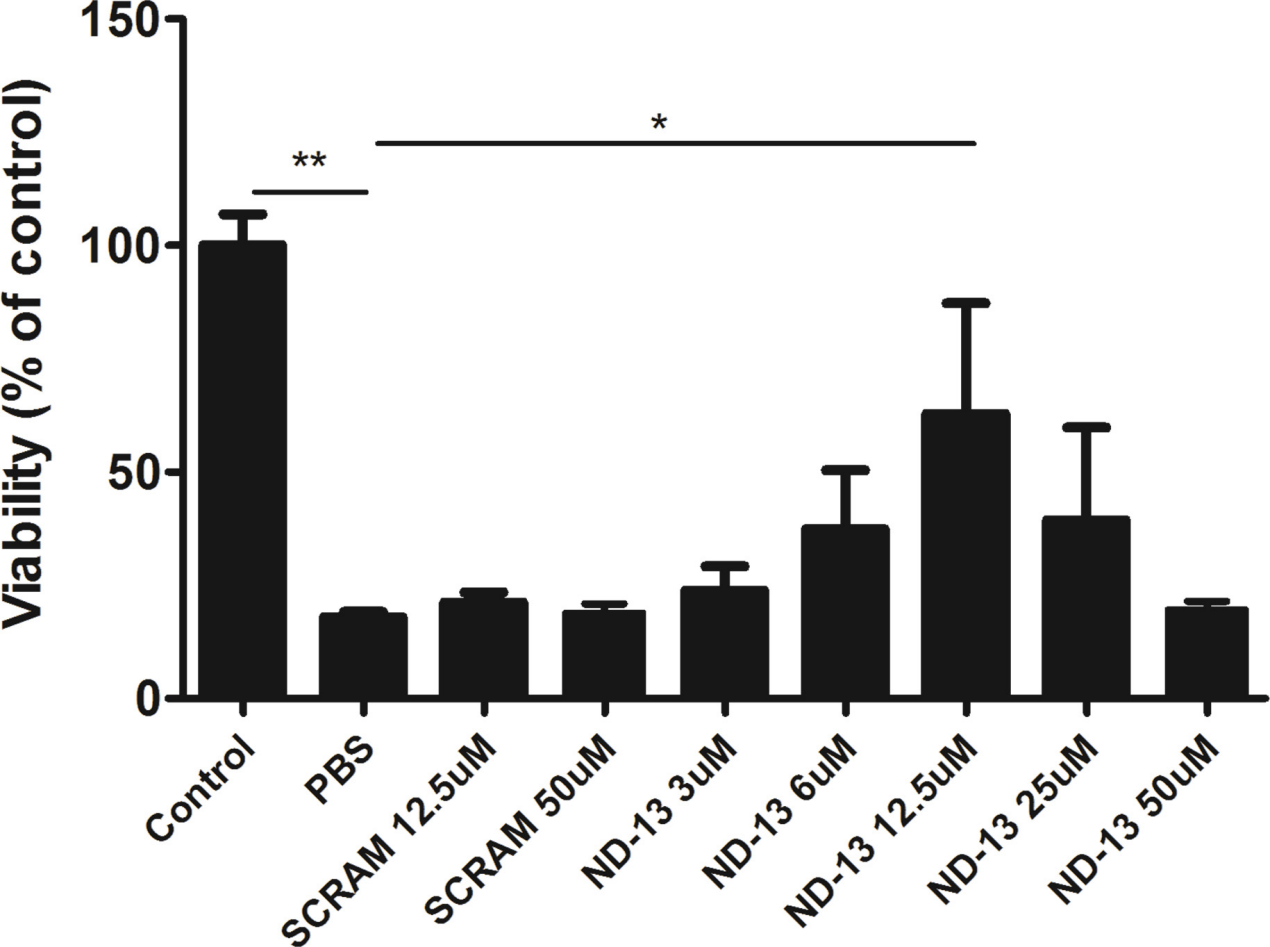
ND-13 protects PC12 cells against the toxic effect of 3-NP. PC12 cells were treated with PBS, ND-13 or a scrambled peptide. One hour later cells were exposed to 3-NP and incubated for 24 hours. Cell viability measurements revealed a significant increase in cell viability after ND-13 treatment (12.5 μM) compared to untreated cells (PBS) and to scrambled peptide. **p< 0.01,*p< 0.05, one-way ANOVA followed by post hoc Dunnett multiple comparison tests. Data is presented as Mean ± SEM.

To evaluate the ability of ND-13 to protect PC-12 cells against 3-NP, cells were treated with increasing concentrations of ND-13 for one hour, followed by exposure to 3-NP (80mM) for 24 hours. Measurements of the cell viability using the Alamar blue assay showed that 3-NP exposure reduces the cell viability to 17.9% (PBS) as compared to untreated cells (Control). Scrambled peptide treatment had no effect on cell viability at 12.5 and 50 μM (21% ± 2.21% and 19% ± 1.89%, respectively). In contrast, 12.5 μM ND-13 significantly increased cell survival, up to 62.83% (p<0.05). ND-13 at higher concentrations showed no additional protective effect. The data were analyzed with a one way analysis of variance (one-way ANOVA) followed by post hoc Dunnet multiple comparison tests (Fig 1).

### ND-13 Protects the Mitochondrial Membrane Potential in Cell Culture

**Fig 2:**
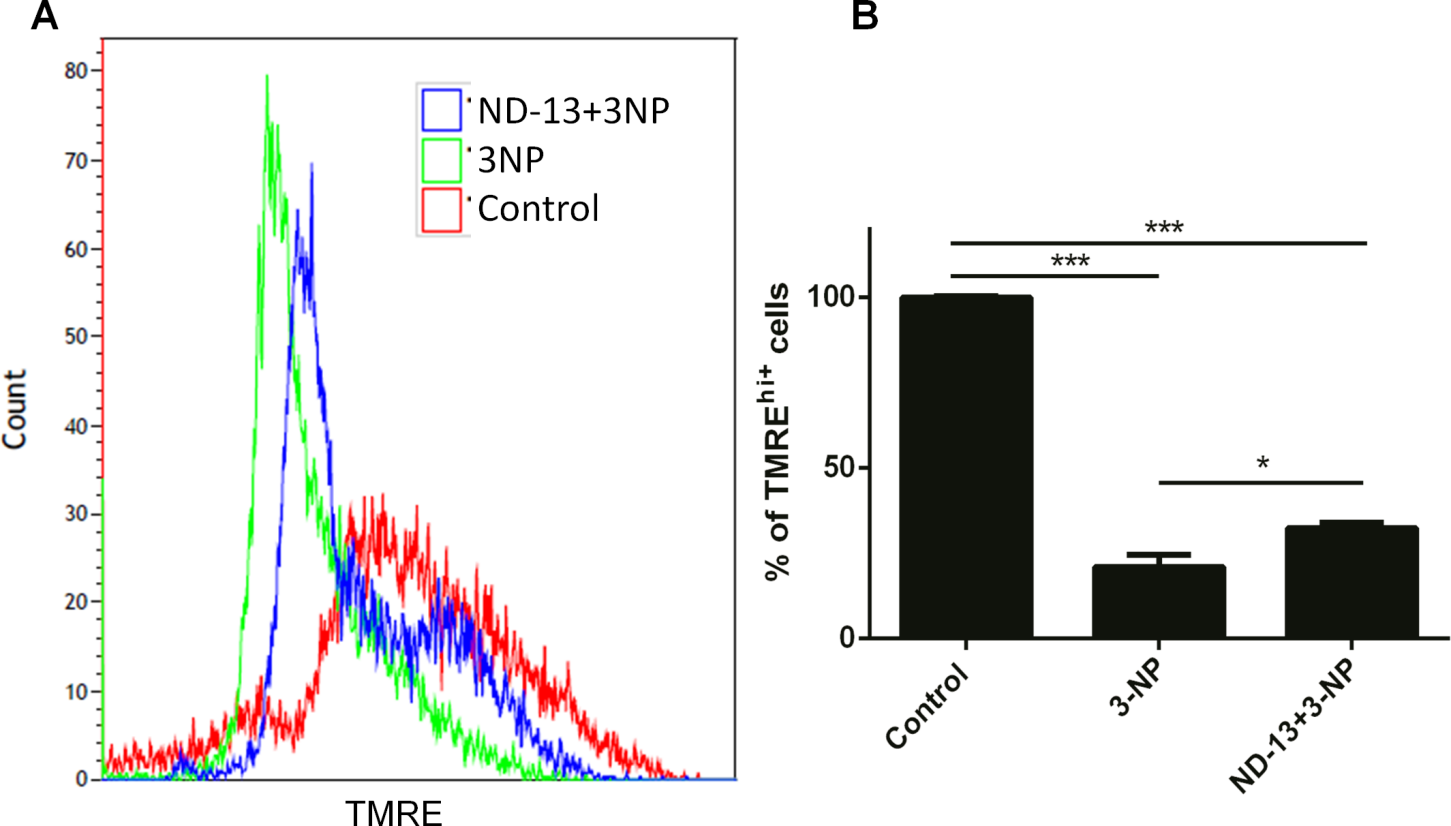
ND-13 improves mitochondrial function in 3-NP treated PC12 cells. PC12 cells were treated with ND-13 or PBS for 1 hour and exposed to 3-NP for further 5 hours. TMRE method was used to evaluate the mitochondrial membrane potential. The intensity of TRME fluorescence of untreated PC12 cells and following treatments with 3-NP +/- ND-13, is presented as Flow histogram (A). The mean percentage of treated cells with high fluorescence intensity was calculated (B). ***p<0.001,*p<0.05, one-way ANOVA followed by post hoc Dunnett multiple comparison tests. Data is presented as Mean ± SEM).

To evaluate the effect of ND-13 on mitochondrial membrane potential and mitochondrial function, TMRE staining was used (see Methods). TMRE is a positively charged red/orange dye that accumulates in active mitochondria. Depolarization or inactivation of the mitochondria decreases membrane potential which leads to reduction in dye fluorescence. Flowcytometry analysis demonstrates that 3-NP-treated PC12 cells showed lower levels of TMRE fluorescence, compared to untreated cells (control), while co-treatment with ND-13 partially preserved TMRE fluorescence (Fig 2A). The fraction of cells expressing high fluorescence intensity (TMRE^hi^) was lower after exposure to 3-NP (22.8%), as compared to control. In contrast, the fraction of TMRE^hi^ cells treated with 3-NP and ND-13 was increased to 32.2% (p<0.05; Fig 2B). These data indicate that the protective effect of ND-13 against 3-NP induced toxicity is via mitochondrial preservation.

### ND-13 Improves Motor Functions in the Toxin-Induced Mouse Model of MSA

We established, for the first time, the 3-NP induced MSA model in mice. Mice injected with 3-NP were compared to mice injected with 6-hydroxidopamine (6-OHDA), a known model of hemi-parkinsonism. First, mice were tested for forelimb asymmetry using the cylinder test [30]: motor dysfunction of the forelimb, contralateral to the injected hemisphere, was observed both, in mice treated with 3-NP and in mice treated with 6-OHDA (S1 Fig). In addition, mice were tested for presynaptic vs. postsynaptic damage of the dopaminergic system via the drug induced rotational behavior. In the amphetamine-induced rotation test, ipsilateral rotations were observed both in 3-NP and 6-OHDA exposed mice, indicating damage to dopaminergic terminals (S2A Fig). In contrast, in the apomorphine-induced rotation test we observed contralateral rotations in mice injected with 6-OHDA, but ipsilateral rotations in mice injected with 3-NP (S2B Fig). This suggests that while striatal injection of both 6-OHDA and 3-NP lead to dopaminergic cell loss, exposure to 3-NP is also accompanied by striatlal cells degeneration at the locus of injection. Thus, we can conclude that 3-NP injection in this mouse model damages both dopaminergic and striatal cells, as seen in MSA patients.

**Fig 3:**
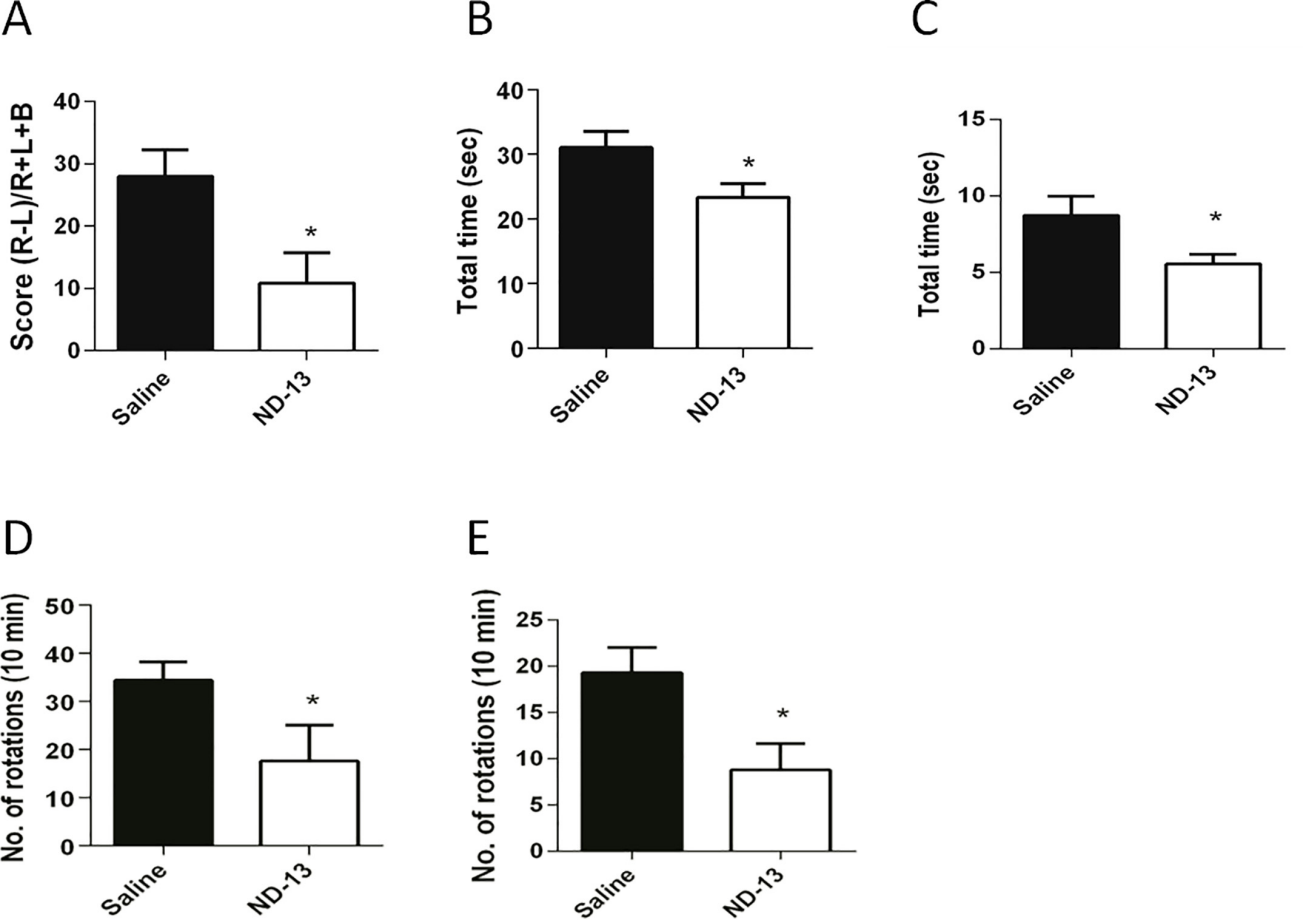
ND-13 attenuates behavioral deficits in 3-NP-induced mouse model of MSA. Motor function and motor asymmetry were measured by the cylinder test, R: right forelimb, L: left forelimb, B: Both forelimbs (A), elevated bridge (B), pole test (C), amphetamine (D), and apomorphine (E). *p< 0.05, t-test, error bars indicates SEM (ND-13: n = 14, Saline: n = 13).

ND-13 was subcutaneously injected twice a day for 5 days to evaluate its ability to protect against 3-NP toxicity. At day 7, the frequency of use of the ipsilateral (unaffected) and contralateral (affected) forelimbs during rearing was measured by the cylinder test. The more frequent use of the ipsilateral forelimb (unaffected) was evident in the saline treated mice (control). However, ND-13 treated mice showed a significant increase in the use of the affected forelimb (Fig 3A) and a reduction of 15% (p<0.05) in motor asymmetry in comparison to the control group.

Next, motor coordination and body balance were evaluated using the elevated beam and pole tests. The time to cross an elevated narrow beam was significantly shorter in mice treated with ND-13, compared to the control group (ND-13: 23.3 sec ± 2.1 sec; Ctrl: 30.08 sec ± 2.5 sec; p<0.05; Fig 3B). A similar improvement was observed in the pole test where mice treated with ND-13 showed a decrease in the time taken to turn their head and body downwards and to descend a pole (ND-13: 5.54 sec ± 0.62 sec; Ctrl: 8.73 sec ± 1.26 sec; p<0.05, Fig 3C).

Striatal and dopaminergic cell depletion due to 3-NP toxicity increases the asymmetry between the brain hemispheres, inducing rotational behavior following administration of amphetamine or apomorphine. Mice with striatal and nigral degeneration rotate in the ipsilateral direction to the locus of insult after being administered either amphetamine or apomorphine (see S2 Fig). The total number of ipsilateral rotations was calculated by subtracting the ipsilateral from the contralateral turns to the lesioned hemisphere (see Materials and Methods). Our results show a significant reduction in the number of ipsilateral rotations in ND-13-treated mice compared to the control group. Following administration of either amphetamine or apomorphine the number of ipsilateral rotations was reduced by half when compared the control, saline-treated group (Apomorphine: ND-13: 11.08 ± 3.4; Ctrl: 20.14 ± 4.6; Amphetamine: ND-13: 17.64 ± 7.4; Ctrl: 34.43 ± 3.7; Fig 3E). Mice injected with saline into the striatum, instead of 3-NP, do not show any asymmetric rotational behavioral (data not shown).

### ND-13 Reduces Inflammation in the 3-NP Injected Mice

**Fig 4:**
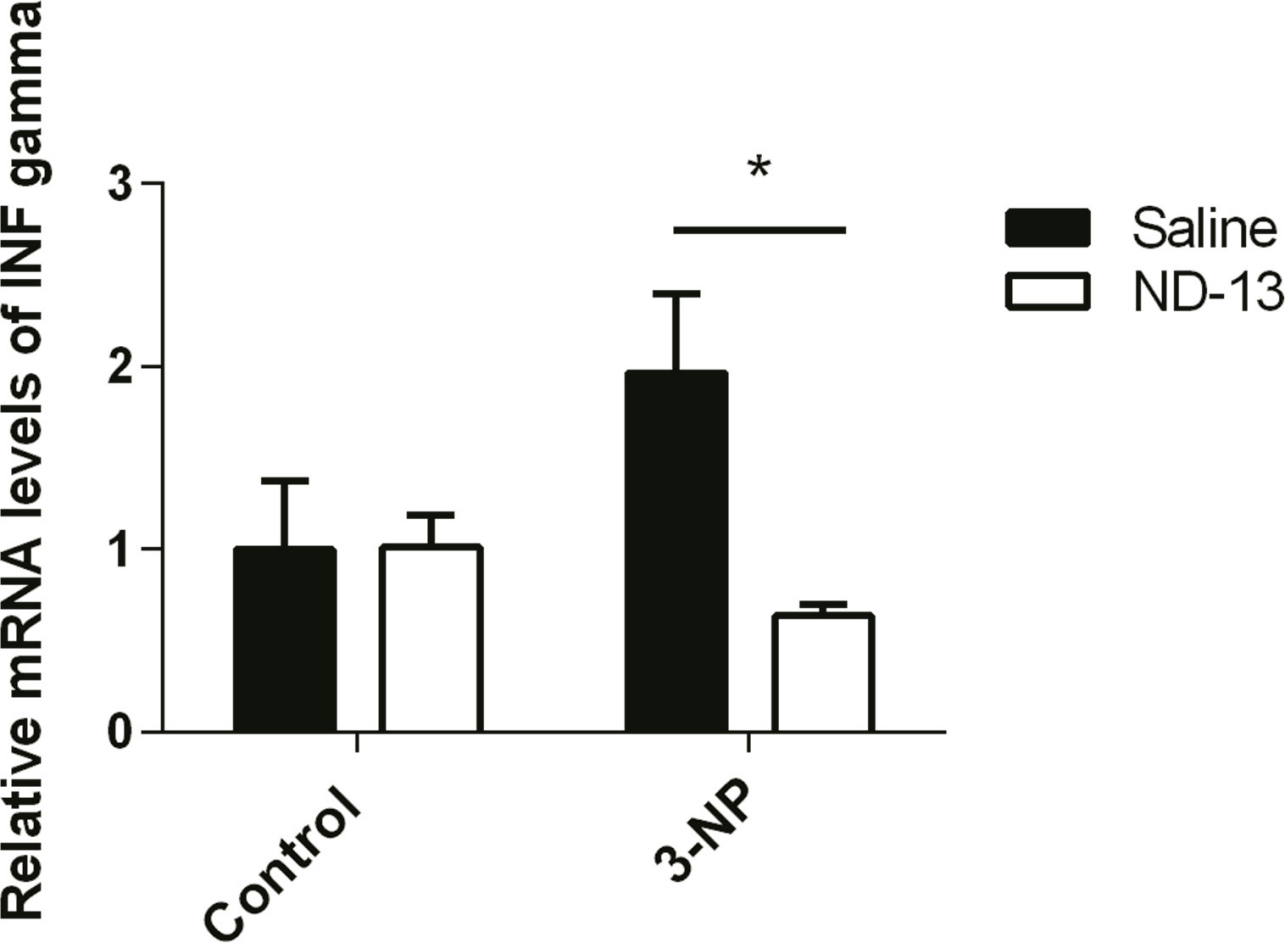
INF gamma mRNA in 3-NP induced mouse model of MSA. To examine the mRNA levels of INF gamma, real-time PCR was conducted on the mice brains. After the treatment with saline or ND-13, the left striatum, not-injected with 3-NP, was used as a control group and was compared to the right striatum, injected with 3-NP. *p< 0.05, two-way ANOVA followed by post hoc Dunnett, error bars indicate SEM (n = 4).

To examine possible ameliorating effects of ND-13 on inflammation, the levels of INF-gamma were analyzed. On the last day of ND-13/saline administration (day 5), four mice of each group were sacrificed and both the right (affected) and left (unaffected) striatum were removed. RT-PCR measurements revealed a significant increase in the expression of INF-gamma after 3-NP injection. However, the INF-gamma values of ND-13-treated mice decreased significantly and they showed similar levels to those of unaffected brain sites (Fig 4).

### ND-13 Reduces Astrogliosis in the 3-NP Injected Mice

**Fig 5:**
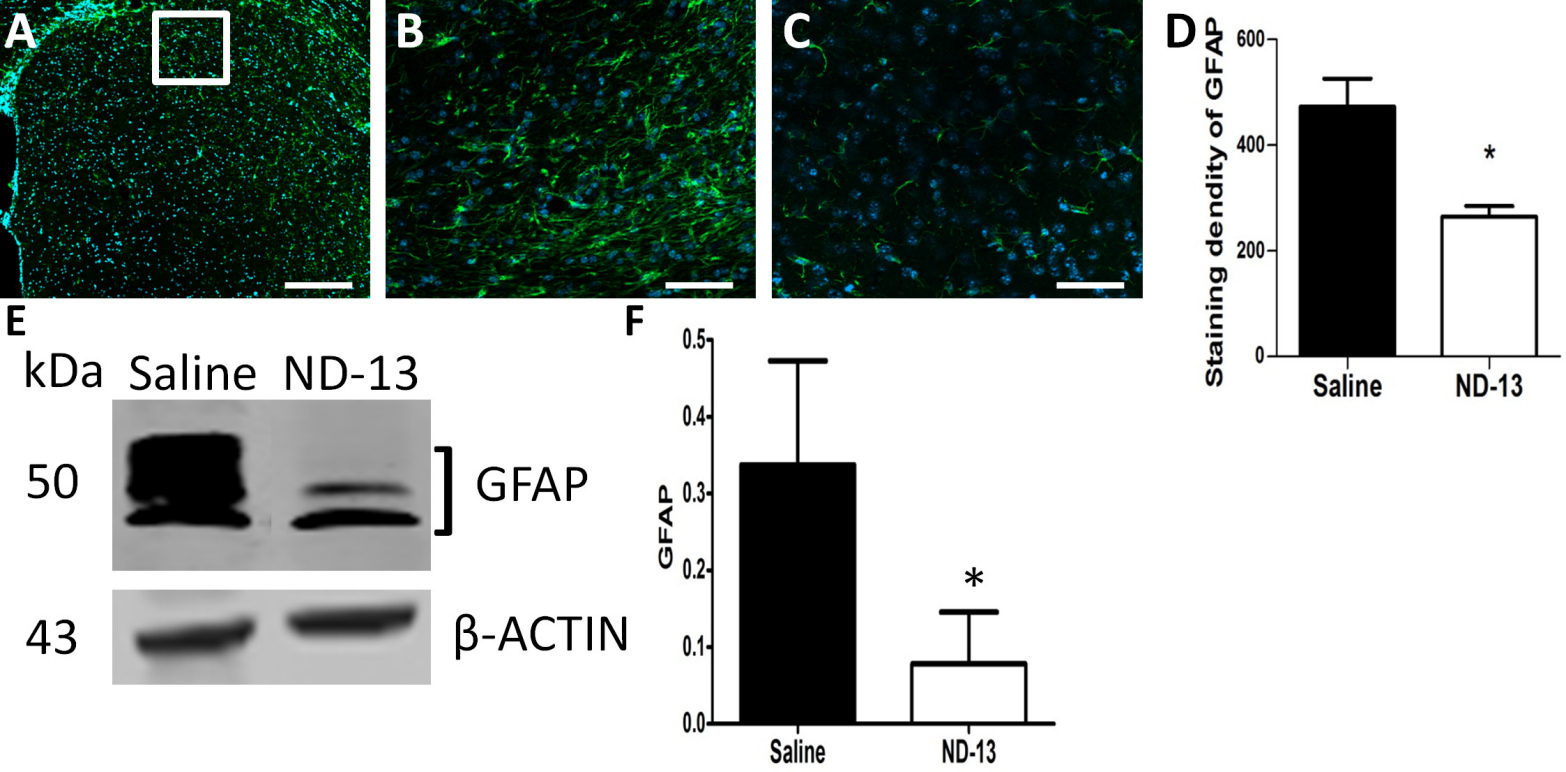
ND-13 reduces astrogliosis in 3-NP injected mice. 3-NP was injected into the striatum (A). GFAP immunostaining demonstrated astrogliosis in mice treated with 3-NP (saline, B) (B). In contrast, mice treated with 3-NP + ND-13 showed a significant reduction in the levels of astrocyte activation (C). The intensity of the staining was calculated by Image-J software (D, *p< 0.05, t-test, error bars indicate SEM, n = 4). Scale bars (A): 200 μm, (B and C): 100 μm. Astrogliosis reduction, following ND-13 treatment was also demonstrated by Western blot analysis with the anti-GFAP antibodies (E and F). *p<0.05, t-test, error bars indicate SEM (n = 4).

Immunofluorescence analysis was performed twenty days after 3-NP injection and after the behavioral tests. Staining for astrocytes by anti-GFAP antibodies revealed strong reactivity in the site of 3-NP injection (473 ± 53%, n = 4). Lower levels of GFAP expression were observed in mice treated with ND-13 (265 ± 19%, n = 4, p<0.05), indicating a reduction in astrocyte reactivity following the treatment (Fig 5A–5C). This was further validated by using Western blot analysis of the proteins extracted from the brain tissue (Fig 5E and 5F).

### ND-13 Preserves Striatal and Dopaminergic Cells in 3-NP Injected Mice

**Fig 6:**
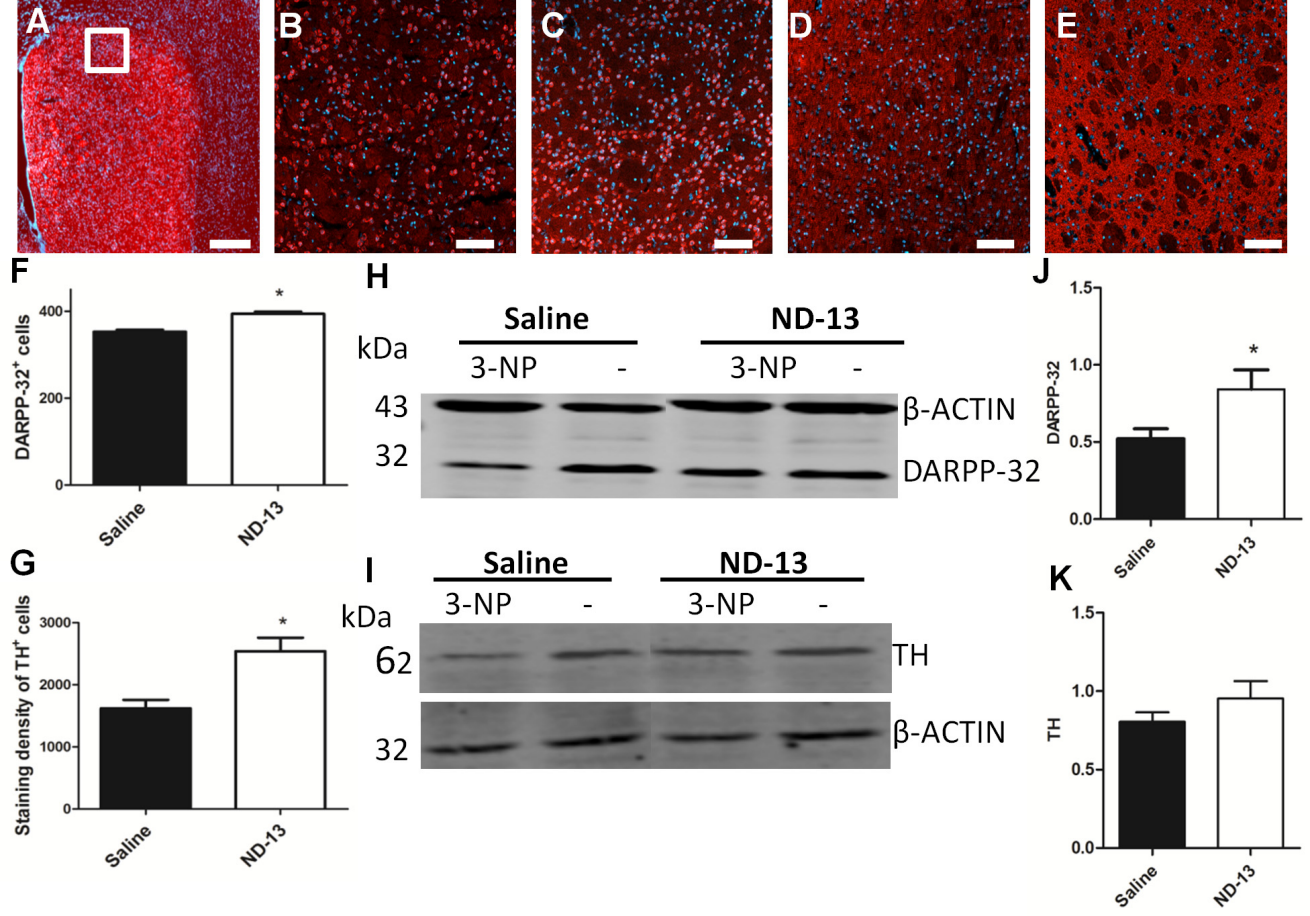
ND-13 preserves striatal cells and dopaminergic terminals in 3-NP injected mice. Immunohistology of brain sections with anti-DARPP-32 antibodies indicates for specific staining in the striatum (A). Mice treated with 3-NP + saline (B, D) and mice treated with 3-NP + ND-13 (C, E) were stained with antibodies against DARPP-32 (B, C) and TH (D, E). The number of DARPP-32 positive cells (F) and the intensity of TH staining (G) were calculated by Image-J software. *p< 0.05, t-test, error bars indicate SEM (n = 5). Scale bars (A): 500 μm and (B-E): 100 μm. DARPP32 and TH levels were also measured by immunoblot analysis (H, I) in the right (3-NP injected) and the left striatum of mice systemically treated with ND-13 or saline. The ratio between left and right hemispheres are shown in J and K.*p<0.05, t-test, error bars indicate SEM (n = 4).

To examine whether ND-13 protects striatal and dopaminergic cells against the toxic effect of 3-NP, we performed immunostaining and Western blot analysis twenty days after 3-NP injection. The immunohistochemical study revealed a significant increase in the levels of DARPP-32 labeling in mice treated with ND-13 compared to saline treated mice (Fig 6A–6C and Fig 6F). Similarly, a decrease in the density of dopaminergic afferents was found after 3-NP injection; the decrease was reversed after treatment with ND-13 (Fig 6D and 6E and Fig 6G, p<0.05).

Western blot analysis revealed a decrease in the striatal specific protein, DARPP-32 to 52%, and a decrease in the dopaminergic specific protein, TH to 80% in the 3-NP-injected hemisphere, compared to the intact hemisphere (Fig 6H and 6I). We found that ND-13 treatment significantly restored the levels of DARPP-32 in the 3-NP-injected striatum up to 84% (Fig 6J, p<0.05). We also observed an increase in the levels of TH, to 95%, although it didn’t reach statistical significance (Fig 6K).

## Discussion

MSA is a prevalent neurodegenerative disease which is characterized by extensive striatal and nigral neuronal loss. The present study describes the effect of treatment with a short DJ-1-based peptide, ND-13, on a toxin-induced mouse model of MSA, which mimics the pattern of cell loss, observed in MSA patients. To this end we have assessed the contribution of the peptide to cell and mitochondria viability *in-vitro* and also to performance of animals in motor-related tasks, following administration of the mitochondrial inhibitor 3-NP. We demonstrate that ND-13 protects cells and rescues mitochondrial function from the toxic effect of 3-NP. Furthermore, subcutaneous administration of ND-13 for a period of five days reversed the 3-NP induced dopaminergic and striatal neuronal loss and ameliorated motor disability in mice treated with the peptide.

3-NP is a mitochondrial toxin which inhibits succinate dehydrogenase activity affecting several areas of the brain [31]. In various animals models it has been shown that 3-NP induces oxidative stress in the striatum, leading to neurotoxicity and neurodegeneration [32], [33]. The specific mechanism of striatal lesion by 3-NP was demonstrated by Kim el al. (2000), showing that 3-NP led to neuronal death by glutamate toxicity followed by oxidative stress. Waldner et al. [34] showed that a single intrastriatal injection of 3-NP, in rats, produces local striatal cell loss as well as retrograde loss of dopaminergic cells in the substantia nigra, while intraperitoneal (IP) injections produce degeneration in the striatum only. Here we demonstrated similar effects after intrastriatal injection of 3-NP in a mouse model. Moreover, using transgenic mice models of MSA, it was shown that IP injection of 3-NP enhances motor deficits and neurodegeneration in nigral-striatal system [10], [11].

DJ-1 is a multifunctional protein that is involved in diverse biological processes. DJ-1 participates in transcriptional activation antioxidative stress response, and mitochondria regulation [35]. In response to oxidative stress, DJ-1 regulates the activity of several transcription factors. One of them is the nuclear factor erythroid-2 related factor (Nrf2). Nrf2 is a transcription factor involved in the regulation of numerous genes associated with anti-oxidant and anti-inflammatory response [36], [37]. In our previous study, we demonstrated that ND-13, a peptide derived from DJ-1, activates Nrf2, up-regulates antioxidant genes and provides neuroprotection in Parkinson's disease mouse models [27].

Another key function of DJ-1 is in maintaining mitochondrial homeostasis [38], [20]. In normal physiological conditions, DJ-1 is localized in the cytosol, mitochondria, and nucleus [39]. Under oxidative stress conditions, DJ-1 is translocated from its major cytosolic pool to the mitochondria, providing cell protection against oxidative agents [40]. Depletion of DJ-1, leads to alterations of mitochondrial morphology, fragmentation, decreased mitochondrial membrane potential, and reduced ATP levels [38]. In the current study, we examined the effect of ND-13 against the toxic effect of 3-NP. We found that ND-13 preserves cell viability and mitochondrial membrane potential in a cell culture. Thus, we can speculate that one of the mechanisms by which ND-13 protects cells is by stabilizing the mitochondrial function.

In order to assess the pathological effect of ND-13 on mice, behavioral tests and biochemical experiments were performed. We observed that mice treated with ND-13 had fewer motor deficits. These findings were consistent with a decrease in dopaminergic and striatal cell degeneration. Moreover, real time PCR analysis revealed that 3-NP administration increases the levels of INF gamma. This finding is in accordance with other studies showing that 3-NP induces inflammation, by increasing proinflammatory cytokines and gliosis [10], [41]. We found that ND-13 significantly reduces the levels of mRNA INF gamma, indicating that ND-13 also decreases inflammation in the 3-NP induced mouse model of MSA.

In conclusion, our data propose DJ-1 as a novel therapeutic target for MSA and suggest ND-13 as a candidate drug for neuroprotection.

## Supporting Information

S1 Fig Motor deficits in mice injected with 3-NP or 6-OHDA. In the cylinder test the use of the non-impaired forepaw (right forepaw) is expressed as percentage of the total use of both paws (R: right forepaw, L: left forepaw, B: both forepaws). We found a significant increase in the use of the non-impaired forelimb after 3-NP or 6-OHDA injection, indicating that injection of 3-NP and 6-OHDA cause motor deficits in mice.
(TIF)

S2 Fig Striatal and nigral degeneration in mice injected with 3-NP or 6-OHDA. (A) Amphetamine induced rotation was tested four weeks after 3-NP and 6-OHDA lesion. Ipsilateral rotations were observed in both 3-NP and 6-OHDA treated mice. (B) Apomorphine-induced rotation was assessed two weeks after 3-NP or 6-OHDA injections. Mice injected with 3-NP showed ipsilateral rotation whereas mice injected with 6-OHDA showed contralateral rotation.
(TIF)
