# Supplementary figures and images for: Neuroprotective Effect of a DJ-1 Based Peptide in a Toxin Induced Mouse Model of Multiple System Atrophy
Source: PLoS One. 2016 Feb 22;11(2):e0148170. doi: 10.1371/journal.pone.0148170 (PMC4763099; doi:10.1371/journal.pone.0148170)

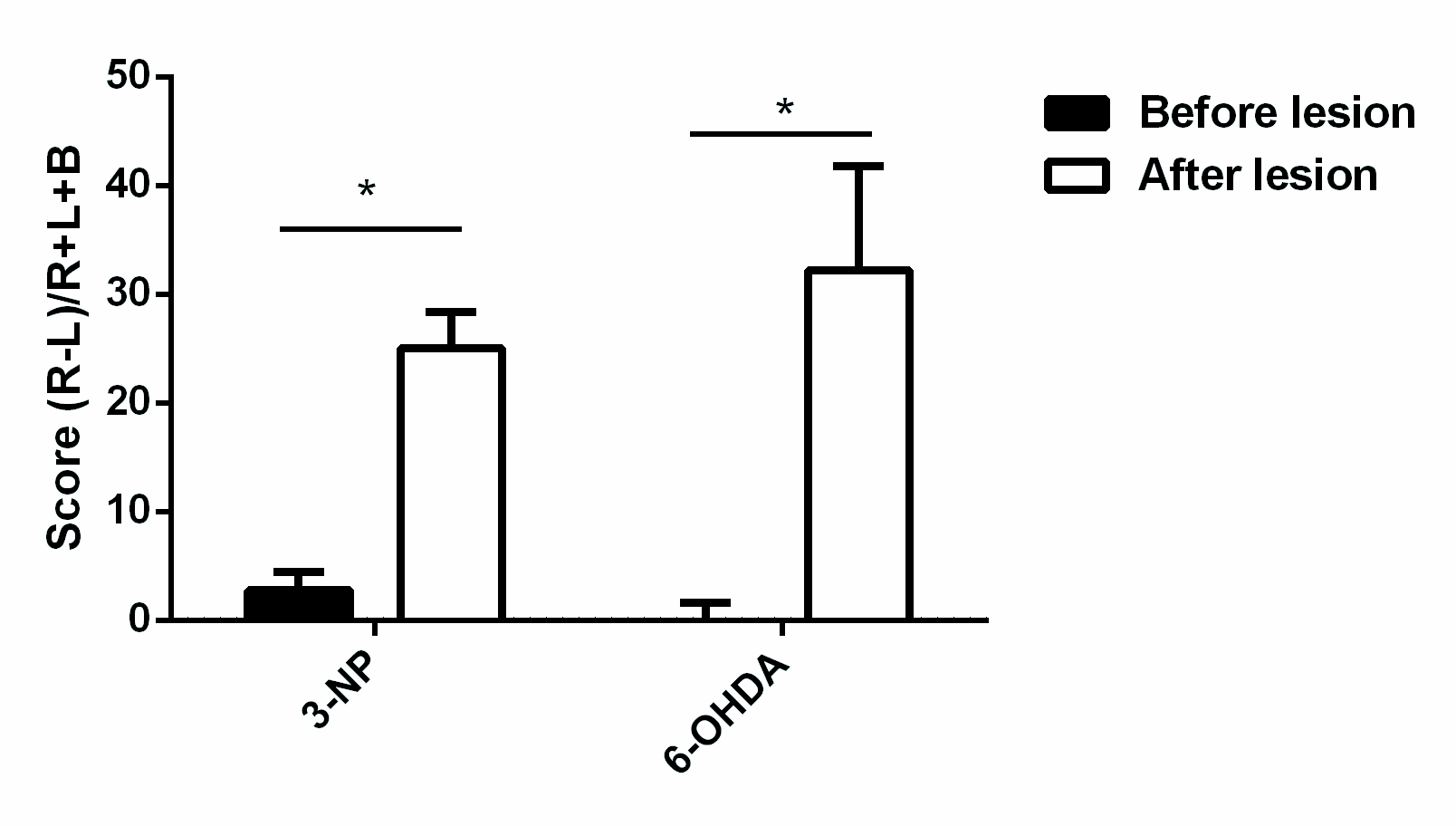

Supplement: S1 Fig — In the cylinder test the use of the non-impaired forepaw (right forepaw) is expressed as percentage of the total use of both paws (R: right forepaw, L: left forepaw, B: both forepaws). We found a significant increase in the use of the non-impaired forelimb after 3-NP or 6-OHDA injection, indicating that injection of 3-NP and 6-OHDA cause motor deficits in mice. (TIF) [file pone.0148170.s001.tif]

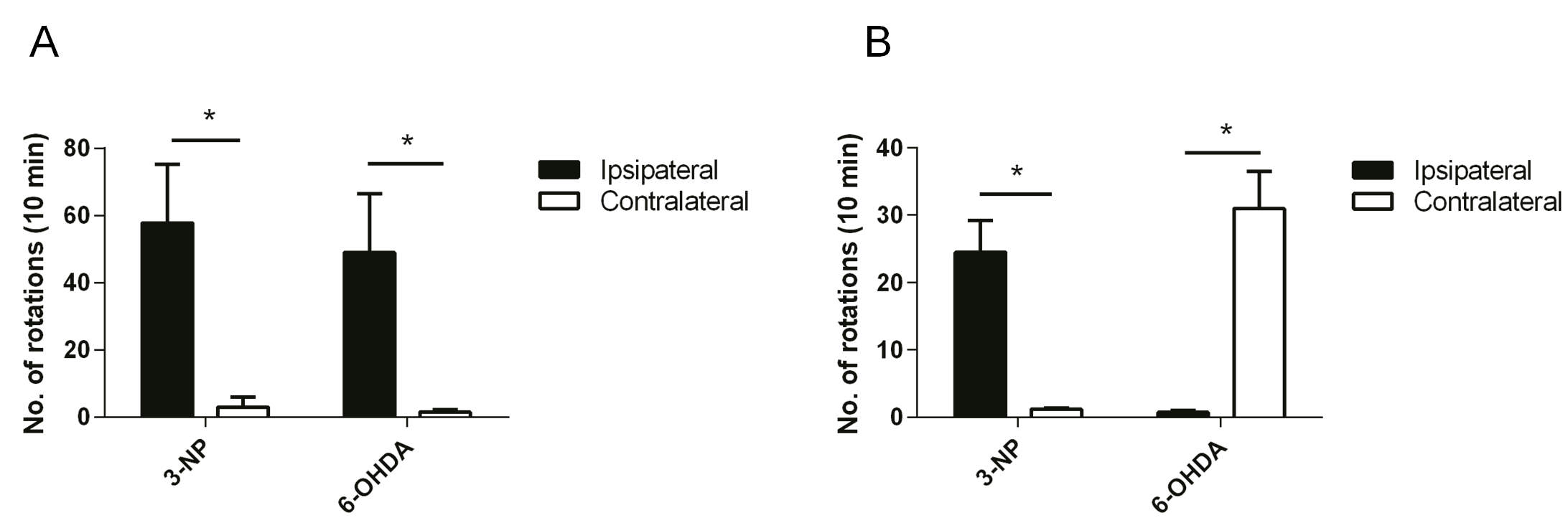

Supplement: S2 Fig — (A) Amphetamine induced rotation was tested four weeks after 3-NP and 6-OHDA lesion. Ipsilateral rotations were observed in both 3-NP and 6-OHDA treated mice. (B) Apomorphine-induced rotation was assessed two weeks after 3-NP or 6-OHDA injections. Mice injected with 3-NP showed ipsilateral rotation whereas mice injected with 6-OHDA showed contralateral rotation. (TIF) [file pone.0148170.s002.tif]
